# Clinical Humanities in Primary Care for Year 2 Medical Students: A Student perspective

**DOI:** 10.15694/mep.2018.0000193.1

**Published:** 2018-09-04

**Authors:** Preeti Sandhu, Ann Wylie, Niki Jakeways, Liza Kirtchuk

**Affiliations:** 1 King's College London

**Keywords:** Clinical Humanities, General Practice, Longitudinal Placement, Medical students' curriculum

## Abstract

This article was migrated. The article was marked as recommended.

As part of a new MBBS Curriculum at GKT Medical School, King’s College London, second year medical students undertook a clinical humanities assignment during their longitudinal GP placement. Groups of students all produced a humanities output relating to medicine and patient care in the community. This article explores the experiences from the student perspective and key learning points for subsequent cohorts, identifying four themes from the feedback obtained in a student evaluation: Broadening horizons; teamworking and leadership, wider community care involvement and seeing patients through different eyes.

## Introduction

Medical undergraduate curricula in the United Kingdom (UK) are guided and regulated by the General Medical Council to ensure that qualifying doctors are suitably prepared and have demonstrated the knowledge, skills and behaviours required for safe practice in their foundation years ( General Medical Council, 2018). Curricula developers have significant responsibilities in balancing core and optional content in an already “crowded curricula” with the challenge as how to incorporate new contents for the evolving healthcare issues in the 21 ^st^ Century ( Ten Cate, 2014). The introduction of clinical humanities in medical education demonstrates a push by medical schools to enhance students understanding of the patient perspective to healthcare, as well as developing compassionate doctors ( Gillies, 2018) ( Wass, 2018). Clinical humanities in medical education in the United States is more developed than in the UK and has seen courses in literature and painting included as part of the medical syllabus, with arts being mandatory in over 50% of American medical schools ( Banaszek, 2011). Emerging interest in medicine and humanities in the UK has been suggested to be a response to the shortcomings of the more traditional scientific, evidence-based approaches to medicine ( Greaves, 2000). Recent advancements in the UK has seen the development of the Association for Medical Humanities, with many universities now encompassing arts with healthcare within their undergraduate medical course ( Association for Medical Humanities, 2018).

As part of a new MBBS Curriculum, second year medical students at GKT Medical School, King’s College London, undertook a General Practice (GP) longitudinal placement. Students spent one day per week, in groups of 8-14 at a GP placement in London, totalling 24 days during the academic year. The placement consisted of a range of activities from regular teaching time in clinics, visiting patients at home and a clinical humanities assignment. The concept of clinical humanities was introduced to the students as a group assignment as part of their programme during this placement.

The aim of the clinical humanities task was for students to engage with a different approach to their learning that was beyond the boundaries of a traditional medical degree. School based support and guidance for the humanities assignment was based on 2 half-day workshops and faculty updates. Students were advised to contact the faculty for further guidance as they progressed with the task, such as queries about ethical approval and consent. This article focuses on the students’ perspective of the clinical humanities project. Data are taken from the end of year student evaluation completed by 64% (N=242) of students at the end of the placement, with free text coded and analysed using NVivo 11.

## Results

### Emerging Themes

**Figure 1.**
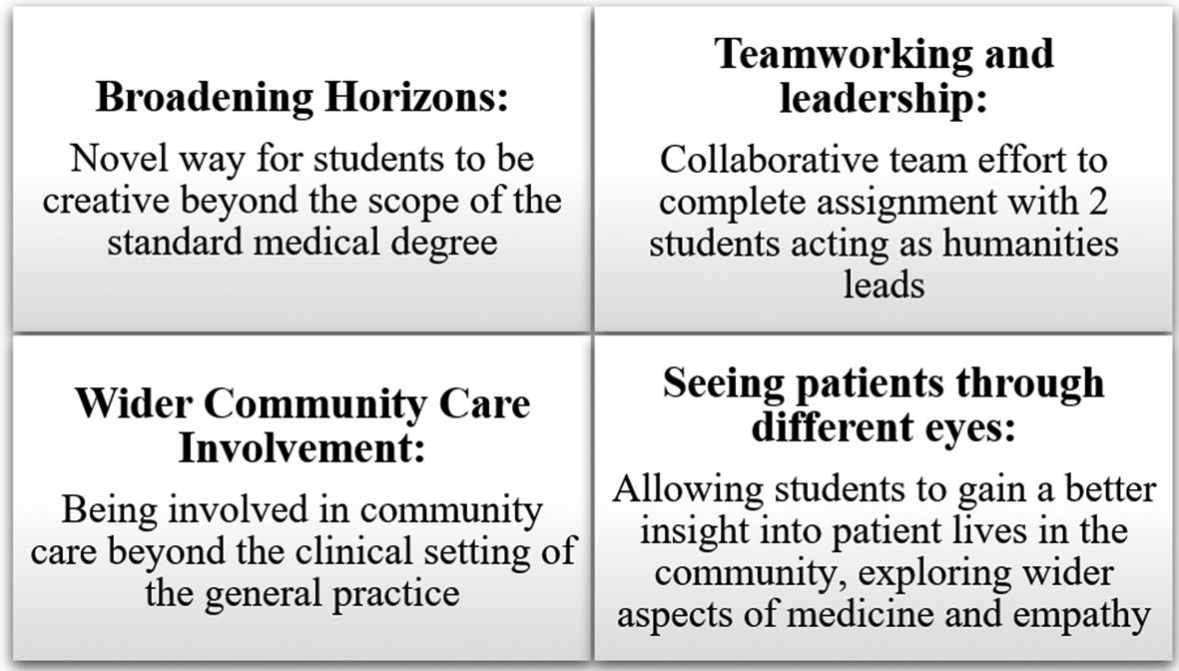
Clinical Humanities task: Four themes identified from the student evaluation

Feedback obtained from students identified four key themes as shown in Figure 1. Each theme is discussed in further detail below.

***Broadening Horizons:*** The flexibility of the task encouraged students to “think outside the box” whilst encompassing arts and humanities, with the scope to delve creatively. The freedom in creating this clinical humanities output provided the opportunity for students to explore a different style to learning, away from the perceived boundaries of the taught course. The continuity inherent in the longitudinal placement allowed for some student groups to create a humanities outputs that was of benefit to the general practice (e.g. health promotion video for patients in the waiting area). Students described enjoying creating something useful for their general practice, able to see it in use, and found this helped develop their understanding of patient care. Many students commented that the experience was enjoyable and on the *“fun”* aspect to this part of their intense medical curriculum course, finding it worthwhile and a *“break”* away from the intense clinical aspect of their medical curricula.

*“Helped understand the meaning of healthcare from an artistic perspective” (student response from the student evaluation)*

*“I really enjoyed this novel way to learn through creativity” (student response from the student evaluation)*

***Teamworking and Leadership:*** Two students per group led on the humanities project and hence had the opportunity to develop their leadership skills. Some of the humanities leads commented on the difficulties of this, finding the responsibility of dividing the workload equally amongst peers and motivation from certain members a challenge.

*“As a project lead, I feel the humanities project was a great way to broaden our horizons and should be continued, however, I feel there should be more emphasis on everyone in the group having to play a part, as it was very much left to the leads devices at times, which is not fair in regards to the rest of the workload we all have.” (student response from the student evaluation)*

Many students enjoyed working as a team pointing to the benefits of collaborative learning, researching the local GP community and working with patients.

*“It was a great way to explore the humanity behind medicine and really brought students and clinicians together” (student response from the student evaluation)*

*“Good to explore aspects outside of the curriculum and work collaboratively as a team.” (student response from the student evaluation)*

***Wider Community Care Involvement:*** Some students developed an interest in the local community, resulting in assignments that focused on this or on targeted patients, as well as wider social issues such as exploring homelessness and access to healthcare by interviews and illustrations of patient belongings.

*“Initially I was sceptical about the usefulness of this project, but at the end I was able to see the point of it. Through this project, I learnt a lot about myself, not only as an individual, but as a member of the team. I also gained a deeper appreciation for the Practice’s role in the community that it serves.” (student response from the student evaluation)*

***Seeing patients through different eyes:*** Students were exposed to the diverse health and social issues faced within London general practices, leading to a greater experience of issues affecting patients, such as difficulties in access to healthcare and patient concerns. Students also visited patients at home to understand better how patients coped with conditions away from the clinical environment and this inspired some to focus their project on their learning from this.

*“Made me look at the patient experience with new eyes.” (student response from the student evaluation)*

*“The project got us to explore patient perceptions and to experience the GP surgery through the eyes of the patient which was an invaluable experience.” (student response from the student evaluation)*

## Challenges

***More guidance and support:*** This was the first year of a new MBBS curriculum, a longitudinal GP placement and of the clinical humanities assignment. Students were provided with an introductory session and booklet detailing learning outcomes for the assignment, as well as the 2 humanities student leads attending campus-held sessions for specific project support and feedback. The task involved creativity from students to generate an idea and implement it together as a team without direct faculty supervision, which posed a challenge for some. Some students felt the objective and purpose of the group assignment was not clear in comparison to clinical tasks required for the progression of their placement. Feedback from placement GP tutors similarly showed a desire for more guidance, especially to support students more effectively (GP Evaluation data).

*“Good project but felt like we were thrown into the deep end without guidance, but I could see the potential value behind it.” (student response from the student evaluation)*

***Creative vs clinical:*** The main challenge for some students was that the delivery of the clinical humanities task involved creativity, with many unable to see the link between arts, humanities and clinical practice. Whilst this challenged students into abstract thinking, others questioned the relevance of the task and its alignment with the core medical curriculum. Some felt that time would have been better spent in teaching clinics at their GP or practising examination skills. With the deadline of the assignment coinciding with other coursework, students found that the clinical humanities assignment did take up a significant amount of time and coordination that was not always possible during placement hours.

*“I am not a creative person, and I found this project challenged my perceptions and pushed me to grow and develop throughout.” (student response from the student evaluation)*

*“I understand the point of the project, however it took up a lot of time that we could have spent in the GP surgery with patients practising our skills and building our medical knowledge.” (student response from the student evaluation)*

**Figure 2.**
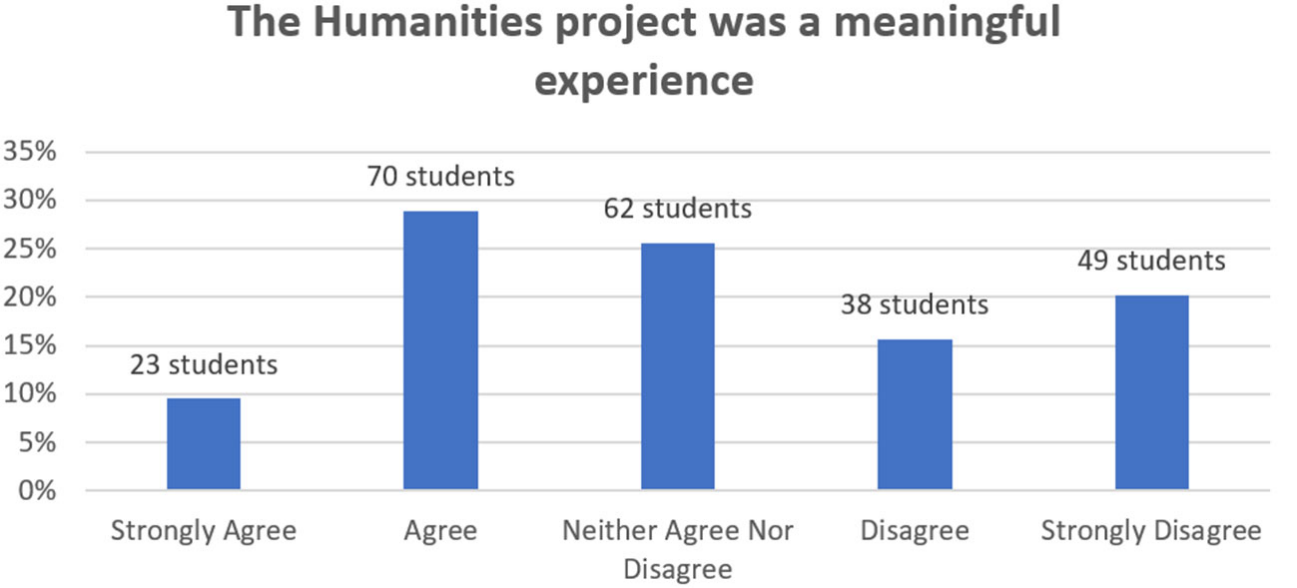
Clinical Humanities task: Student evaluation responses about the meaningfulness of the humanities assignment.

Data from the student evaluation in Figure 2 shows the variation in responses when students were asked how meaningful they found the humanities task, with 38% either strongly agreeing or agreeing they found it meaningful and 35% either strongly disagreeing or disagreeing that they found it meaningful.

## Discussion

The challenging findings obtained from the student evaluation illustrate the need for more clarity from the medical school regarding the planning and delivery of the clinical humanities task. Providing students with more information at the start of the academic year may have increased students’ confidence in understanding what was expected. Whilst the clinical humanities task aimed to encourage medical students to learn in a self-directed approach, more guidance is still a prerequisite for self-directed learning to be of benefit, as well as the development of clear learning objectives, identifying appropriate resources and an educator as a facilitator ( Murad and Varkey, 2008)

The concept of arts and humanities with medicine was interpreted by many students as a requirement to be a creative individual or familiar with arts, expressing more value in time spent in consultations with patients or learning clinical skills. Medical students undertaking humanities courses have been shown to take it less seriously than the rest of their curriculum, with a possible explanation being that the freedom of expression with the clinical humanities task resulted in some students being unable to contextualise what they had learntfrom humanities into the clinical setting ( Wachtler, Lundin et al., 2006). With humanities often being hard to define and not the evidence-based medicine that students are used to being taught, difficulties lie in both the student’s perceptions towards arts in science and the challenge of curricula developers in teaching so ( Jones, Kittendorf et al., 2017). More support for students on what the humanities task entails, as well as examples of previous clinical humanities outputs will benefit the next student cohort on their understanding of expectations of the task.

Some students found that although initially sceptical about the purpose of the clinical humanities task, they were able to immerse themselves fully into creating something of benefit to the community or their general practice as they were able to see the value of humanities in the context of enhancing patient centred awareness, community service and population support. A recent study in the United States found that medical students who engage in humanities were more empathetic and had greater emotional intelligence, suggesting that humanities helps students become better doctors with positive personal qualities ( Mangione, Chakraborti et al., 2018) *,* as well as the use of arts encouraging medical students to explore illness and medical care in a patient-centred approach ( Jones, Kittendorf et al., 2017). However, unless medical students can see the relevance clinically of a topic, their enthusiasm and motivation in that topic may be diminished as a result ( Lam, Irwin et al., 2002), which may explain why some students did not find the humanities assignment as meaningful or as useful to their medical education.

Despite an increase in responsibility on the clinical humanities leads for each project, students became more aware of their leadership and organisational skills. Opportunities for medical students to develop leadership and management competencies have been promoted by the NHS with the development of the Medical Leadership Competency Framework, yet the requirement for leadership learning is not part of the core curricula in many medical schools ( NHS Institute for Innovation and Improvement and Academy of Medical Royal Colleges, 2010). Whilst second year medical students are still in the early stages of their medical career, being able to progress in their training as leaders and advocate change in the clinical setting are skills that can be still be explored early on, providing the basis for future career progression.

Students also commented that they struggled to find the balance between various assignment deadlines and the completion of the clinical humanities assignment. With the demands of the medical course increasing each year, introducing humanities in second year whilst on placement was deemed the most appropriate time as students were already in groups based in the same location weekly, thus the continuous setting provided regular contact time between peers.

## Conclusion

The introduction of clinical humanities in the second-year medical curricula at GKT Medical School received mixed responses from students. Students engaging well with the clinical humanities task were able to see the benefit of health and social care in the community and developed an increased level of understanding of issues patients encounter. To ensure more active engagement from all students, clearer learning objectives and guidance should be provided to students and GP tutors alike as well as more support from the medical school. Retaining the clinical humanities task in second year is justifiable due to students being placed on a longitudinal placement providing continuity of teamwork and a familiar community setting as a base.

## Take Home Messages

• Clinical humanities should be introduced early in the medical curriculum to ensure active engagement from students in arts, humanities and patient-care
• Clinical humanities tasks encourages students to be involved with community care and population-level public health
• Second year medical students at GKT Medical School needed more support and guidance to aid in their development of the clinical humanities task with clearer learning objectives
• Students need encouragement to see the benefit of arts in medicine alongside their traditional clinical and medical skills learning

## Notes On Contributors

Preeti Sandhu is a third year medical student studying at King’s College London.

Dr Ann Wylie leads on core Global health teaching and health promotion in King’s undergraduate medical curriculum for many years, is deputy director of the community medical undergraduate curriculum and senior teaching fellow. She has presented related research at many international conferences.

Dr Niki Jakeways is a GP and the Phase 3 Community Lead Assistant at King’s College London

Dr Liza Kirtchuk is a GP and the Stage 2 General Practice Lead for the longitudinal GP placement at King’s College London.
