# Supplementary material for: Clinical Humanities in Primary Care for Year 2 Medical Students: A Student perspective
Source: MedEdPublish (2016). 2018 Sep 4;7:193. [Version 1] doi: 10.15694/mep.2018.0000193.1 (PMC10701805; doi:10.15694/mep.2018.0000193.1)
Supplement: Peer review history

# Reviewer report: Reviewer response for version 1

**DOI:** 10.21956/mep.19650.r29302

**Published:** 2019-03-14

**Authors:** Richard Hays

**Affiliations:** 1 James Cook University

This review has been migrated. The reviewer awarded 3 stars out of 5 I enjoyed reading this paper. As a curriculum designer, teacher, family doctor and passionate art lover, I need no encouragement to offer medical students opportunities to understand the relationship between medical practice and the rest of life. It has also been my experience and observation that such strategies work well for many (not all) junior medical students, but how to continue this through the senior years appears much more difficult. There are some weaknesses in the paper that the authors should considre in future writing. There is a rather bumpy transition from introduction to results, which could have been avoided by having a heading and a paragraph on the method. Some suggestions for adopting similar strategies elsewhere may also be useful in helping others to avoid any obvious pitfalls.

Reviewer Expertise:

NA

No decision status is available

# Reviewer report: Reviewer response for version 1

**DOI:** 10.21956/mep.19650.r29301

**Published:** 2018-09-05

**Authors:** Raquel CORREIA

**Affiliations:** 1 SOS Médecins Paris

This review has been migrated. The reviewer awarded 4 stars out of 5 An interesting article on a current theme describing an innovative way of integrating humanities into the pregraduate curriculum. I liked the clear way the reader is guided in the introduction and the use of relevant sources.I would, however, like to see more information about the content of the workshops that are made prior to the activity as well as how the leaders of each group were chosen and whether there was a specific realm or definition of objectives for these roles.In the results, it would be interesting to know how the 4 themes were chosen by the authors.It seems to me relevant to note that there is a kind of duality between those who found this project relevant and those who found it useless. As a matter of curiosity, it would be interesting to have an attachment with the demographics of the participants and the answers to the questionnaire.Overall, I think the article is really interesting, especially given that we have the perspective of a student. Thanks to the authors for the work and implication.

Reviewer Expertise:

NA

No decision status is available

# Reviewer report: Reviewer response for version 1

**DOI:** 10.21956/mep.19650.r29303

**Published:** 2018-09-04

**Authors:** Trevor Gibbs

**Affiliations:** 1 AMEE

This review has been migrated. The reviewer awarded 4 stars out of 5 A very interesting paper describing a novel approach to introducing the Humanities to a group of very young medical students. I liked the way that the activity also introduced other aspects of learning- leadership, teamwork etc.I particularly liked the way that the authors placed the Humanities task into the four themes. For me, this could have been made somewhat clearer by providing examples of what the students actually did during this time, what was the collective activity of the students.There was an interesting dichotomy amongst the students as the the perceived value ot the task, I think that this might have been worth exploring through either deeper interviews or some background demography.Of course the value of this teaching hopefully manifests itself through how it shapes the student for the future, so I hope that the authors consider a longitudinal study to follow these students further into their learning journey.

Reviewer Expertise:

NA

No decision status is available
